# Successful retrosternal esophageal bypass with gastric transposition in the management of esophago-pleural fistula as a complication of Boerhaave syndrome

**DOI:** 10.1016/j.ijscr.2023.108797

**Published:** 2023-09-09

**Authors:** A. Piratheepan, S. Inthujan, V. Sutharshan

**Affiliations:** Department of Surgery, Teaching Hospital Jaffna, Sri Lanka

**Keywords:** Boerhaave syndrome, Esophago-pleural fistula, Esophageal bypass, Gastric pull-up, Gastric transposition, Case report

## Abstract

**Introduction and importance:**

Boerhaave syndrome is a rare life-threatening condition that represents about 15 % of esophageal perforation and is associated with significant mortality. A subset of patients with effort rupture of the esophagus can present with esophago-pleural fistula. Management of esophago-pleural fistula remains a challenge due to the lack of high-quality evidence studies and the rarity of reported cases. Esophageal bypass with gastric transposition could have a role in management by using the same principles used in chronic esophago-pleural fistula in esophageal malignancy.

**Case presentation:**

We report a unique case of a 33-year-old male with effort rupture of esophagus who developed esophago-pleural fistula successfully managed with an esophageal bypass with gastric transposition after multiple attempts of CSES placement have failed.

**Clinical discussion:**

Boerhaave syndrome is a rare clinical presentation with mortality ranging from 20 to 50 %. A case of Boerhaave syndrome present with esophageal pleural fistula is uncommon despite the anatomical proximity of these structures. For delayed presentation deployment of CSES to control the fistula was not effective as retrosternal esophageal bypass in this case study.

**Conclusions:**

Esophageal bypass with gastric transposition might be an effective strategy for esophago-pleural fistula compared to CSES placement following delayed presentation of Boerhaave syndrome but further appropriately designed studies are required to make recommendations.

## Highlights

• The management of esophago-pleural fistula as a complication of Boerhaave syndrome
• Retrosternal Esophageal bypass with gastric transposition was safe and effective in management of esophago-pleural fistula
• Further clinical research is needed before routine use in clinical practice.

## 1 Introduction

Effort rupture of the esophagus, also known as Boerhaave syndrome is uncommon, first described in 1724, by Hermann Boerhaave, transmural perforation of the esophagus resulting from a sharp increase in intraluminal pressure against closed cricopharyngeal sphincter due to neuromuscular incoordination during forceful vomiting usually following overindulgence of alcohol/food. It represents about 15 % of esophageal perforation with incidence of 3.1 per 1,000,000 per year [1,2].

Although it classically presents by the Meckler triad of chest pain, vomiting, and subcutaneous emphysema these are not always present. Mortality of Boerhaave ranges from 20 to 50 % [3,4], often due to delay caused by the nonspecific nature of symptoms as there are no classical symptoms and presentation can mimic many other medical conditions.

Management depends on size, location, and associated complications with perforation as well as comorbidities of the patient and baseline status of the esophagus, though nonoperative management has been the preferred approach in a substantial number of patients, surgery is the mainstay of treating the effort rupture of the esophagus and it's complications, however, their routine use in the contexts of an esophago-pleural fistula has not been established [5,6], this may be the first case of esophageal bypass in the context of Boerhaave syndrome complicated with esophago-pleural fistula.

We discuss a case of delayed presentation of effort rupture of the lower thoracic esophagus to the tertiary care hospital, where initial endoscopic therapy with Covered Self Expanding Stent placement (CSES) failed and successfully managed with an esophageal bypass with gastric transposition.

This case report has been reported in line with the SCARE Criteria [7].

## 2 Presentation of case

**Fig. 1:**
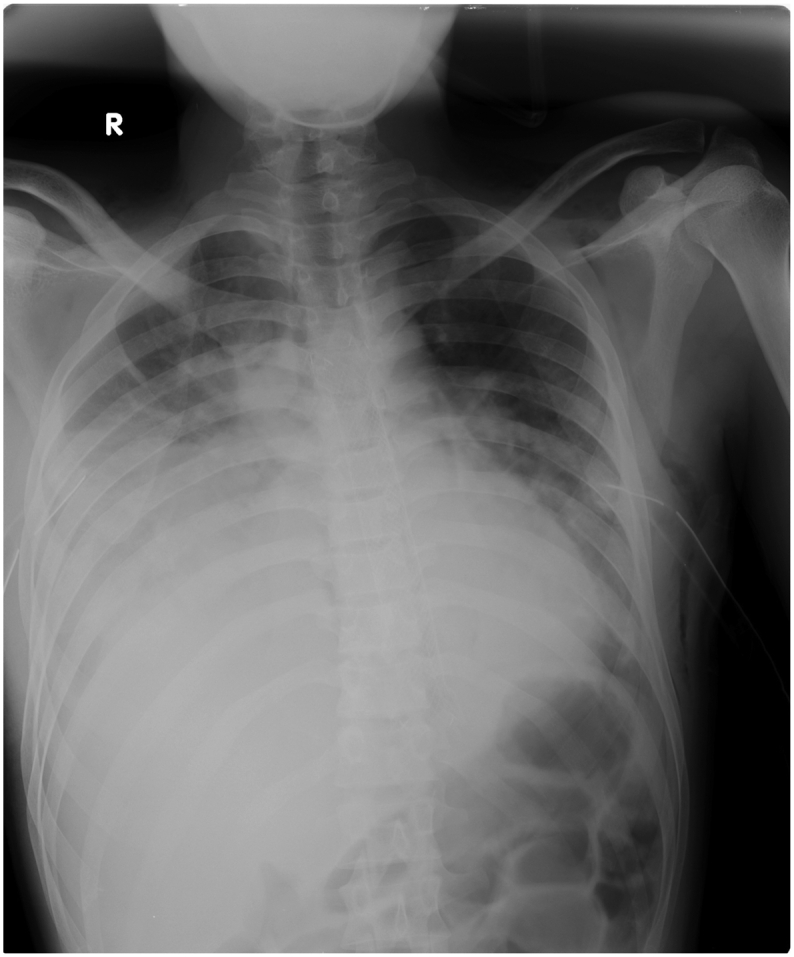
Chest radiograph showing right-sided pleural effusion and pneumomediastinum.

**Fig. 2:**
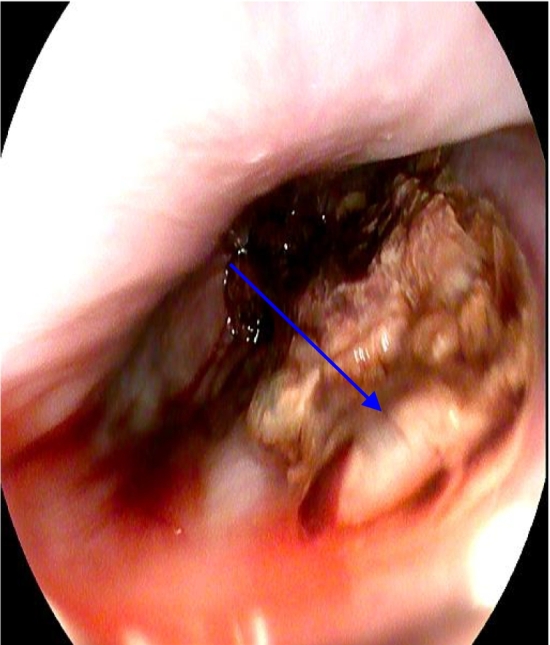
A longitudinal tear in the lower esophagus with unhealthy edematous edges marked with an arrow.

33-year-old previously apparently well presented to the emergency treatment unit of a nearby community hospital with sudden onset lower chest pain and shortness of breath after one episode of hematemesis following a bout of alcohol intake on the previous day. Furthermore, he had 3 more episodes of hematemesis before admission (volume ∼850 cc). On physical examination, he was pale, blood pressure 104/60 mmHg, pulse rate 120/min, saturation 96 % on air with increased work of breathing (WOB), reduced breath sound on the right lower zone, subcutaneous emphysema distributed in the neck and the upper chest, with epigastric tenderness and guarding. His investigation revealed hemoglobin 10 g/dl, C-reactive protein (CRP) 192, and chest X-ray showed bilateral pleural effusion and pneumomediastinum, subcutaneous emphysema (Fig. 1). Furthermore electrocardiograph (ECG) and troponin I were negative. A right-sided chest drain was placed and drained up to 1000 cc of blood. He was transferred to a nearby health care facility with an improved facility for further management after transfusing 1 pint of packed red cells. He underwent a contrast-enhanced computed tomography (CECT) with oral contrast which revealed pneumomediastinum, extensive surgical emphysema in the neck and anterior chest wall, bilateral pleural effusions with adjacent passive atelectasis and no contrast leakage from the esophagus. The patient underwent an upper gastrointestinal endoscopy (UGIE) that was suspicious for perforation of the lower esophagus with slough (Fig. 2).

**Fig. 3:**
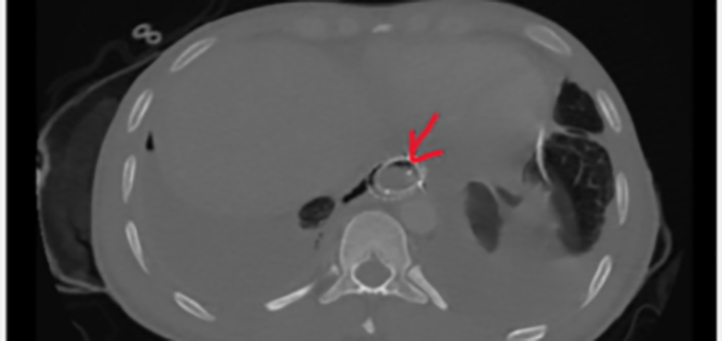
CT-lung window showing two overlapping CSES marked with an arrow.

Later he was transferred to a tertiary care facility for further management. On admission, initial management included nil by oral, intravenous fluids, vitals and input, output monitoring, intravenous antibiotics, proton pump inhibitors, and a left intercostal drainage tube inserted. The patient was admitted to the accident and trauma intensive care unit for monitoring and further care. Chest and limb physiotherapy, DVT (deep vein thrombosis) prophylaxis, and incentive spirometry were undertaken from the time of admission. OGD was repeated on day 1 after admission to tertiary care facility which showed unhealthy mucosa at 35 cm with bubbling noted and a 15 cm anti-reflux covered self-expanding stent (CSES) was placed (Fig. 3).

During the ICU stay he developed worsening SOB and fever spikes (102 °F) on day 4 and antibiotics escalated (clindamycin added to meropenem and metronidazole) and High flow nasal oxygen therapy started, with increasing oxygen demand and WOB ventilatory support given.

**Fig. 4:**
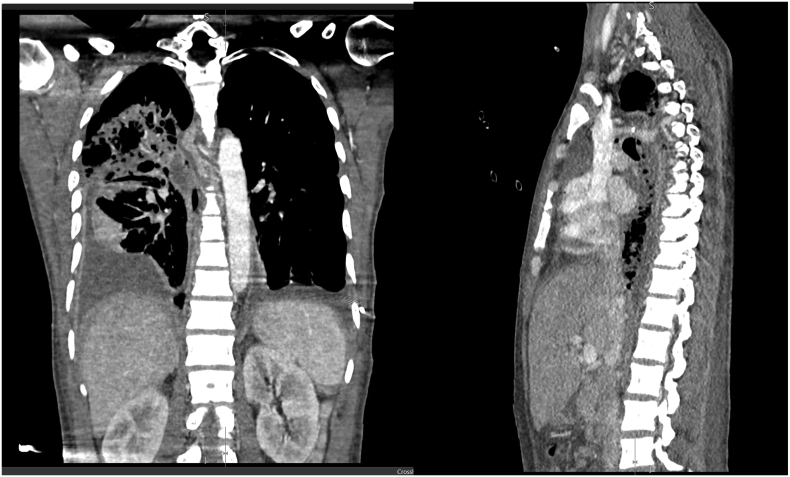
CECT chest showing encysted right-sided pleural effusion with multiple air loculi and periesophageal collection on the right side.

He underwent an HRCT-chest which revealed pneumonia involving 60 %–80 % of lung parenchyma with bilateral encysted effusion (more on the left side) likely because of nosocomial pneumonia. Left-sided encysted pleural effusion was managed with a pigtail drain. He had persistent high output from the Right chest drain containing pale yellow creamy fluid, He underwent a repeat UGIE on day 10 which showed cephalad migration of the stent, and another overlapping CSES was placed. Repeat CECT was performed which revealed interval regression of right-sided and mediastinal collection with no significant change in left-sided encysted effusion. He underwent video-assisted thoracoscopic washout (VATS + washout) on day 14 (Fig. 4).

**Fig. 5:**
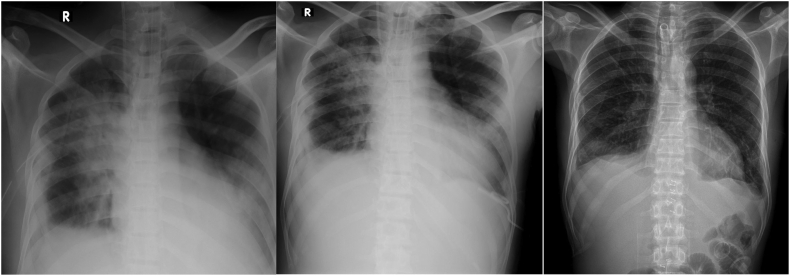
Series of chest radiograph showing improvement following thoracoscopic drainage (VATS) and decortication of the lungs showing.

During the procedure, a moderate amount of serosanguineous fluid and loculated fluid collection drained from the left side and a moderate amount of loculated pus drained from the right side (Fig. 5).

On day 5 (day 27 from the onset) after VATS, a percutaneous endoscopic gastrostomy (PEG) feeding tube was inserted by replacing the nasogastric feeding as we suspected NG tube exaggerated the gastroesophageal reflux, but even after that PEG, feeding was persistently drained through the chest drain. After he was optimized with total parenteral nutrition (TPN) and intravenous octreotide, on day 8 after VATS, he underwent esophageal bypass with gastric transposition, the postoperative period was uneventful. He was improving clinically and biochemically and was discharged without complication on postoperative day 16.

We have done a review of him at intervals of 2 weeks, 4 weeks, and 3 months following discharge, the patient's weight gain, oral intake and wound healing of the surgical site were satisfactory, and serum albumin and hemoglobin have improved significantly.

## 3 Discussion

Boerhaave syndrome is a rare clinical presentation with mortality ranging from 20 to 50 % [5,6]. A case of Boerhaave syndrome present with esophageal pleural fistula is uncommon despite the anatomical proximity of these structures [2]. Effort rupture of the esophagus commonly occurs in the lower third of the esophagus on the left side, 2–4 cm proximal to the cardia of the stomach [8].

The delayed diagnosis of Boerhaave syndrome due to its non-specific symptoms can lead to serious mediastinitis and sepsis. But in our case, chest x-ray revealed surgical emphysema and pneumomediastinum which led to the suspicion of effort rupture of the esophagus, although the positive rate of x-ray findings in the background of Boerhaave syndrome is only 42.9 % [4].

In our case, CECT with esophageal contrast didn't reveal any contrast extravasation from the esophagus, though CT is relatively sensitive for the detection of esophageal perforation. This may be attributed to established edema and muscle spasm because CT was taken 48 h after the initial presentation. Fluoroscopic esophagogram was not performed as gastrografin was not available at the local health care facility.

Endoscopic evaluation has high sensitivity (100 %) and specificity (80 %) [9] but it has its complications such as extending the rupture and worsening the pneumomediastinum with possible contamination [10]. In our case, as the CT showed no contrast leak from the esophagus, we proceeded with endoscopy which revealed a longitudinal tear in the posterior aspect of the lower esophagus. Ideally, the endoscopy should be done by an experienced endoscopist at a specialist center where a stenting facility is available if the perforation is confirmed.

The risk of leakage is 83 % for primary repair for patients presenting after 24 h because of inflammation, empyema, and unhealthy edges [11]. As our patient transferred to the tertiary center after being managed in two different district general hospitals had caused a significant delay (approximately 72 h) in initiating appropriate management. So ideal treatment for patients with late presentation to tertiary care (>72 h) is still under dispute. Conservative treatment mainly includes intravenous antibiotics and placement of chest drains and a few studies revealed some cases have been cured without surgery [12,13].

Even though the recommendation of covered self-expanding stent (CSES) placement is within 48 h, we attempted CSES considering the patient's condition at that time, CSES placement after delayed presentation might be as a good option as neither surgical options nor CSES placement have proven effectiveness for delayed presentation. There was persistent leakage even after CSES with esophageal contents leaking into the chest drain. Failure of CSES is more common than expected in many studies [14]. We attempted a second overlapping CSES, but we were not be able to control the esophago-pleural fistula. It is reported replacement of stents is a feasible approach in anastomotic leak or chronic esophageal fistula, but it does not seem to be promising in treating effort rupture of the esophagus complicated with fistula [15,16].

We attempted Video Assisted Thoracoscopic Washout (VATS + Washout) and decortication of the lung as the patient developed sepsis due to loculated empyema with extensive mediastinal abscess.

Formation after discussion with respiratory and interventional radiology team. VATS is a safe procedure comparable to thoracotomy and washout but minimizing the additional surgical trauma in already very ill patients [17].

As our patient had persistent leak from the perforation and the worsening of the patient's general condition, we considered esophageal bypass with gastric transposition (Gastric pull-up) over partial esophagectomy or delayed repair of the esophagus after optimizing him with TPN and IV Octreotide.

in which the patient was positioned supine, approached via midline laparotomy, gastric conduit created with right gastroepiploic pedicle using LigasSure™ and 100 mm (green) Gastrointestinal anastomosis (GIA) linear cutting staplers. Intra-abdominal esophageal stump with cardia left in situ. In the cervical phase of dissection left cervical incision was made at the anterior border of the Sternocleidomastoid, the cervical esophagus was dissected with the help of a sling and divided distal segment closed with 2-0 vicryl (polyglactin). The retrosternal tunnel was created using blunt dissection techniques and gastric conduit was brought to the cervical incision without rotation, End to end esophagogastric anastomosis using 2-0 vicryl,16 Fr Nasogastric (NG) tube was placed across the anastomotic site and feeding jejunostomy created.

Postoperatively managed with broad-spectrum antibiotics, Jejunostomy feeding, DVT prophylaxis, and vigorous physiotherapy. Nasogastric (NG) feeding started on post-op day 10, oral feeding was initiated on post-op day 12 patient discharged on post-op day 16 without complications.

## 4 Conclusion

This case suggests that esophageal bypass with gastric transposition for the management of esophago-pleural fistula might be an effective strategy over the endoscopic CSES placement. But other endoscopic strategies such as endoscopic vacuum therapy are currently under evaluation with promising outcomes, with current evidence surgical management is superior to other conservative management in the context of Boerhaave syndrome complicated with esophago-pleural fistula.

## Consent

Written informed consent was obtained from the patient for publication and any accompanying images. A copy of the written consent is available for review by the Editor-in-Chief of this journal on request.

## Ethics approval

Ethical approval is exempt/waived at our institution.

Ethical clearance not required for case reports in our institution.

## Funding

The authors received no financial support for this case report authorship and/or publication.

## Authors' contributions

Authors AP, SI, VS contributed to collection of information and writing of the manuscript. All authors read and approved the final version for publication.

## Guarantor

Dr. V. Sutharshan.

## Research registration

Not applicable.

## Declaration of competing interest

The authors declare that they have no conflicts of interest.
